# Microdifferential Pressure Measurement Device for Cellular Microenvironments

**DOI:** 10.3390/bioengineering12010003

**Published:** 2024-12-24

**Authors:** Mami Akaike, Jun Hatakeyama, Yoichi Saito, Yoshitaka Nakanishi, Kenji Shimamura, Yuta Nakashima

**Affiliations:** 1 Graduate School of Science and Technology, Kumamoto University, 2-39-1 Kurokami, Chuo-ku, Kumamoto 860-8555, Japan; 221d9281@st.kumamoto-u.ac.jp; 2 Institute of Molecular Embryology and Genetics, Kumamoto University, 2-2-1 Honjyo, Chuo-ku, Kumamoto 860-0811, Japan; jhatakey@kumamoto-u.ac.jp (J.H.); simamura@kumamoto-u.ac.jp (K.S.); 3 Fusion Oriented Research for Disruptive Science and Technology, Japan Science and Technology Agency, 5-3, Yonbancho, Chiyoda-ku, Tokyo 102-8666, Japan; 4 Faculty of Advanced Science and Technology, Kumamoto University, 2-39-1 Kurokami, Chuo-ku, Kumamoto 860-8555, Japan; west-east@mech.kumamoto-u.ac.jp (Y.S.); y-naka@mech.kumamoto-u.ac.jp (Y.N.); 5 Institute of Industrial Nanomaterials, Kumamoto University, 2-39-1 Kurokami, Chuo-ku, Kumamoto 860-8555, Japan; 6 International Research Organization for Advanced Science & Technology, Kumamoto University, 2-39-1 Kurokami, Chuo-ku, Kumamoto 860-8555, Japan

**Keywords:** mechanical force, microenvironment, brain pressure, embryo, microdifferential pressure sensor

## Abstract

Mechanical forces influence cellular proliferation, differentiation, tissue morphogenesis, and functional expression within the body. To comprehend the impact of these forces on living organisms, their quantification is essential. This study introduces a novel microdifferential pressure measurement device tailored for cellular-scale pressure assessments. The device comprises a glass substrate and a microchannel constructed of polydimethylsiloxane, polytetrafluoroethylene tubes, a glass capillary, and a microsyringe pump. This device obviates the need for electrical measurements, relying solely on the displacement of ultrapure water within the microchannel to assess the micropressure in embryos. First, the device was subjected to arbitrary pressures, and the relationship between the pressure and the displacement of ultrapure water in the microchannel was determined. Calibration results showed that the displacement *dx* [μm] could be calculated from the pressure *P* [Pa] using the equation *dx* = 0.36 *P*. The coefficient of determination was shown to be 0.87, indicating a linear response. When utilized to measure brain ventricular pressure in mouse embryos, the fabricated device yielded an average pressure reading of 1313 ± 640 Pa. This device can facilitate the measurement of pressure within microcavities in living tissues and other areas requiring precise and localized pressure evaluations.

## 1. Introduction

Mechanical forces, such as compression, tension, and shear, acting on cells and tissues within the body are known to affect cellular proliferation [1,2,3] and differentiation [4,5]. Additionally, these forces are pivotal in tissue morphogenesis and functional expression [6,7,8]. However, the actual magnitudes and changes in these forces within the body are not well understood. Within the body, these forces do not operate independently but interact with each other. Therefore, it is essential to decompose and quantify each force to gain an in-depth understanding of the mechanical forces at play and their effects. The ability to measure and quantify mechanical forces substantially contributes to research and applications in several fields, including cellular and molecular biology, thereby enhancing our understanding of the fundamental mechanisms governing cellular behavior. In turn, this enhanced understanding can lead to the determination of novel strategies for regulating cell proliferation and differentiation. For instance, understanding the influence of mechanical forces on tissue engineering and regenerative medicine is imperative for formulating approaches to enhance tissue growth and repair within environments that mimic their natural contexts. However, mechanical elements have scarcely been incorporated in forming organoids aimed at applications in regenerative medicine [9,10]. Most organoid applications focus on biochemical factors rather than mechanical stimuli. Therefore, it is highly likely that mechanical information could contribute to the improvement of organoid formation. In disease modeling, providing an appropriate physical milieu is crucial for replicating the pathophysiological conditions of specific diseases. This aids in elucidating the etiology and progression of diseases and informs the development of therapeutic interventions. In drug screening and toxicity testing, assessing cells and tissues in environments closely resembling their physiological conditions enables a more precise evaluation of drug effects and toxicity. Hence, accurate measurement and quantification of mechanical forces will expedite research efforts across various life science disciplines and contribute to the innovation of therapeutic methodologies and a deeper understanding of diseases. These advancements are anticipated to enhance disease prevention, treatment, and overall health maintenance.

Several methods have been proposed for measuring mechanical forces within the body. Techniques have been devised to assess compressive and tensile stresses within cells and tissues by injecting oil droplets or gels and observing their deformation [11,12]. The tensile stress experienced by cells and tissues can be effectively measured via laser ablation, a method that precisely severs tissues with a laser and gauges the resultant instantaneous recoil velocity of cells to evaluate relative tissue stress [13,14,15]. Cells can be cultivated on micropillar arrays, and the deflection of the pillars can serve as an indicator of cellular traction forces [16,17]. Alternatively, traction force microscopy is a technique wherein the displacement of beads in a gel deformed by cellular traction forces is recorded [18]. Moreover, methods such as micropipette aspiration [19,20,21] and atomic force microscopy [22,23] have been devised to measure mechanical forces by applying external loads. However, these techniques predominantly assess the mechanical response of cells; notably, direct measurements of hydrostatic pressure are limited. While the servo-nullifying technique is commonly employed for cellular-scale pressure measurements, it can be influenced by environmental factors such as media ion composition, environmental electrical resistance, and ionic diffusion characteristics [24,25,26,27,28,29]. To address this issue, a microscale pressure measurement method based on the curvature deformation of immiscible fluid/fluid interfaces has been proposed. This method utilizes observed changes in the curvature of the fluid/fluid interface to measure hydrostatic pressure at a length scale of approximately 10 microns [29]. However, since it requires close-range observation of the gas–liquid interface under a microscope, it is challenging to measure pressure within microscale spaces embedded in multilayered tissues.

While these approaches are effective for measuring pressure in cells or aquatic organisms, they become impractical for direct measurements within microscale spaces in the tissues of terrestrial animals, such as mice and guinea pigs. These animals are not adapted to underwater environments, making it infeasible to immerse their tissues in water for pressure measurement. Furthermore, tissue extraction for measurements often disrupts the natural state of the biological system, which is undesirable for achieving accurate in vivo assessments. Therefore, a novel method is needed to enable direct pressure measurements within multilayered tissues under conditions that closely resemble the natural biological environment.

This study presents the development of an economical and straightforward microfluidic device capable of directly measuring pressure in microenvironments in air. This device is unaffected by factors such as media ion composition or environmental electrical resistance. The proposed device is fabricated using simple semiconductor manufacturing processes and operates without the need for electrical measurements, relying solely on the displacement of ultrapure water within a microchannel to determine pressure. Additionally, this device is applicable for measuring pressure within microscale spaces embedded in multilayered tissues, enabling measurements under conditions closely resembling natural biological environments. By eliminating the need for water immersion and functioning effectively in air, this device addresses key limitations of conventional methods, expanding the scope of pressure measurement technologies.

## 2. Materials and Methods

### 2.1. Composition and Working Principle of the Proposed Device

**Figure 1:**
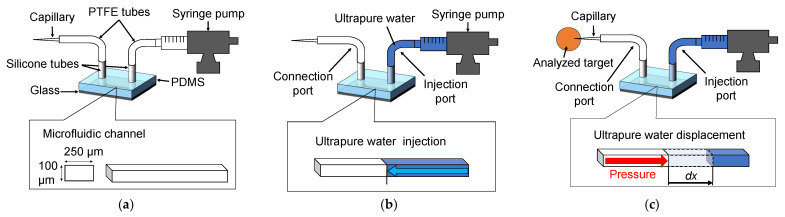
Overview of the microdifferential pressure measurement device. (**a**) Composition of the microdifferential pressure measurement device. The device is constructed from a glass substrate and polydimethylsiloxane (PDMS); the device also comprises a single microchannel, tubes, a capillary, and a micro syringe pump. (**b**) Ultrapure water injection. The microsyringe pump connected to the injection port fills the microchannel with ultrapure water up to half its length. The injection port side is a closed system. (**c**) Ultrapure water displacement. A capillary is attached to the connection port. Upon insertion of the capillary into the analyzed target, the ultrapure water within the microchannel is displaced owing to the pressure within the analyzed target. This displacement is utilized for pressure determination.

The microdifferential pressure measurement device was assembled by utilizing a glass substrate and a microchannel comprising polydimethylsiloxane (PDMS) (KE-106, Shin-Etsu Chemical Co., Ltd., Tokyo, Japan), polytetrafluoroethylene (PTFE) tubes (2-798-02, AS ONE Corporation, Osaka, Japan), silicone tubes (9-869-02, AS ONE Corp.), a glass capillary, and a microsyringe pump (Microinjector IM-9B, NARISHIGE Group, Tokyo, Japan). The schematic of the fabricated microdifferential pressure measurement device is shown in Figure 1. The microchannel cross-section has a width of 250 µm and a height of 100 µm. PTFE tubes were attached to both ends of the microchannel using silicone tubes (Figure 1a). One tube acts as the injection port, whereas the other serves as the connection port. A microsyringe pump was linked to the injection port. Ultrapure water, employed as the working fluid, occupied half the length of the microchannel (Figure 1b). The capillary was connected to the connection port. Upon insertion of the capillary into the analyzed target, the ultrapure water within the microchannel was displaced toward the injection port by the intrinsic pressure of the analyzed target. This displacement was utilized for pressure measurements (Figure 1c).

### 2.2. Device Fabrication

**Figure 2:**
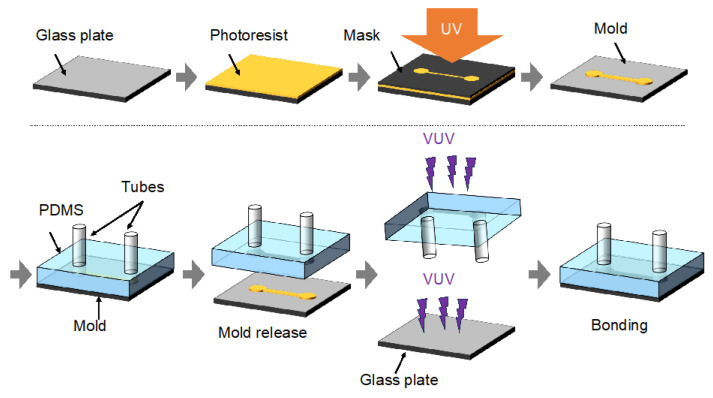
Fabrication process for the microdifferential pressure measurement device. The photoresist was applied to a clean substrate. A mask was then brought into contact with the resist-coated surface and exposed from above. Following exposure, the developed mold was connected to tubes, and PDMS was poured into it. Once the PDMS had solidified, the mold was separated. The PDMS surface and a new glass substrate were irradiated with vacuum ultraviolet (VUV) light and bonded.

The microdifferential pressure measurement device was fabricated by employing photolithographic and soft lithographic techniques (Figure 2). Initially, a silane coupling agent (KBM-402, Shin-Etsu Chemical Co., Ltd.) was applied to a cleaned glass substrate, which was subsequently coated with a negative photoresist (SU-8 3050, Nippon Kayaku Co., Ltd., Tokyo, Japan) using a spin coater (MS-A100, MIKASA Co., Ltd., Tokyo, Japan). This coated substrate was then brought into contact with a photomask featuring the microchannel pattern and exposed to UV light. The microchannel mold was produced using standard photoresist development processes. Silicone tubes were vertically positioned on the fabricated mold, and PDMS was poured in and cured at 70 °C for 1 h. To enhance bonding, the surfaces of the fabricated PDMS microchannels and freshly prepared glass substrates were irradiated with vacuum ultraviolet (VUV) light. This treatment removed organic contaminants from the material surfaces and generated polar functional groups, such as hydroxyl and carboxyl. When these activated surfaces were brought close to each other, hydrogen bonds were primarily formed between the polar functional groups at the interface [30,31]. Subsequently, the interior of the microchannel of the fabricated microdifferential pressure measurement device was coated with a hydrophobic coating (DURASURF, Harves Co., Ltd., Saitama, Japan). The length of the 250 µm by 100 µm microfluidic channel was 13 mm. The silicone tubes had an outer diameter of 2 mm and an inner diameter of 1 mm, with a length of 15 mm at the connection, half of which was buried in PDMS and cured.

### 2.3. Experimental Setup for the Microdifferential Pressure Measurement Device

PTFE tubes with an outer diameter of 1.5 mm, an inner diameter of 0.5 mm, and a length of 400 mm were connected to the silicone tubes at both ends of the microchannel. A microsyringe pump containing ultrapure water was connected to the injection port, and half the length of the microchannel was filled with ultrapure water (1 mL). A glass capillary was attached to the connection port. The capillaries used in the experiment were pulled from glass tubes with an outer diameter of 1 mm and an inner diameter of 0.75 mm (B100-75-10, Sutter Instrument Company, Novato, CA, USA) using a puller (P-97, Sutter Instrument Company), resulting in a tip with an outer diameter of 110 µm and an inner diameter of 100 µm. Additionally, the glass capillaries were polished at an angle of 45° to facilitate penetration through tissue. Furthermore, to evaluate the effect of capillary action, capillaries with and without water-repellent treatment were prepared.

The displacement of ultrapure water within the microchannel, triggered by the insertion of the capillary into the analysis target, was observed using an inverted microscope (TS-100, Nikon, Tokyo, Japan) and documented with an attached camera (X10 Super System, Canon, Tokyo, Japan). Movies capturing the displacement of ultrapure water in the microchannel were subsequently analyzed using the software program ImageJ. The displacement was quantified as the variance in the length of the microchannel occupied by the ultrapure water before and after capillary insertion. The initial position of the liquid surface, before pressure was applied, was set as the zero point. When pressure was applied, the liquid surface moved. At 30 s intervals after the start of pressure application, the positions of the liquid surface were manually marked. Each point’s position was recorded as x and y coordinates and saved in a CSV file. The distances between the marked points were calculated from the x and y coordinates. These distances were defined as the displacement of the liquid surface. A calibration procedure established the correlation between the displacement of ultrapure water and the pressure.

### 2.4. Calibration Method of the Microdifferential Pressure Measurement Device

**Figure 3:**
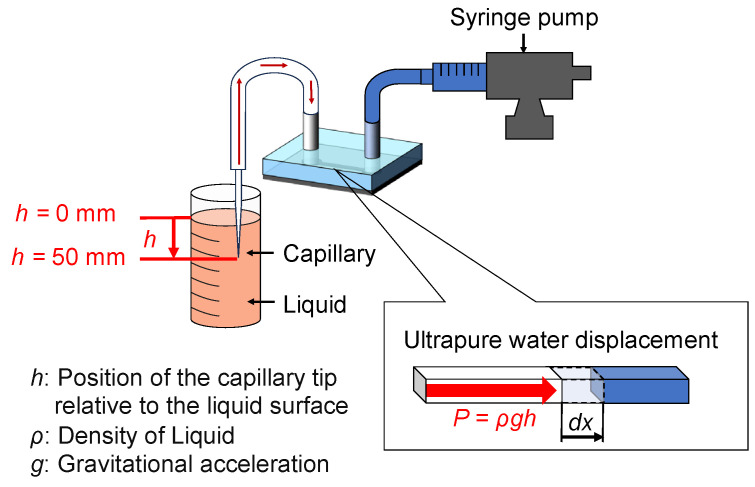
Calibration method of the microdifferential pressure measurement device. The effect of capillary action was evaluated, and a pressure application experiment was conducted by immersing the capillary tip in the liquid. This calibration process also accounts for factors such as air compression, friction, and the elastic deformation of silicone.

Pressure was applied to calibrate the device by adjusting the position of the capillary tip from the liquid surface (0 mm) to a depth of 50 mm, as shown in Figure 3. Fetal bovine serum (FBS) was used as the calibration liquid because cerebrospinal fluid (CSF), which fills the ventricles of a mouse embryo’s brain and is the focus of subsequent pressure measurements, is derived from blood and is thought to contain various serum components. Therefore, FBS was chosen as an appropriate substitute. Additionally, an experiment was performed to evaluate the effects of capillary action. For this, a glass capillary was connected to the microdifferential pressure measurement device, and its tip (1–3 mm) was immersed in ultrapure water for 300 s.

Thus, the relationship between the displacement of ultrapure water and resultant pressure could be established. The relationship between pressure *P* [Pa] and displacement of ultrapure water *dx* [µm] is represented by Equation (1), with the constant c determined through calibration.

(1) dx=cP,

where the pressure *P* [Pa] is calculated using the equation

(2) P=ρgh,

where *ρ* is the density of the liquid, *g* is the gravitational acceleration, and *h* is the height. *h* refers to the position of the capillary tip relative to the liquid surface. The density of ultrapure water was set to 997.1 kg/m^3^, and the density of FBS was set to 1007 kg/m^3^ [32]. The gravitational acceleration was 9.81 m/s^2^, and the height was varied from 0 mm to 50 mm as mentioned earlier. This calibration process also accounts for factors such as air compression, friction, and the elastic deformation of silicone.

### 2.5. Method Used for Lateral Ventricular Measurements

All animal experiments adhered to the facility’s guidelines and were approved by the licensing committee of Kumamoto University (A2021-095, A2022-043, A2023-065).

Brain pressure was assessed in mice. Timed-pregnant mice from the Institute for Cancer Research were procured from Japan SLC (Hamamatsu, Japan), with the day of vaginal plug detection designated as embryonic day 0 (E0). Mouse embryos at stage E12.5, corresponding to the stage when the ventricles were visibly identifiable, were utilized.

**Figure 4:**
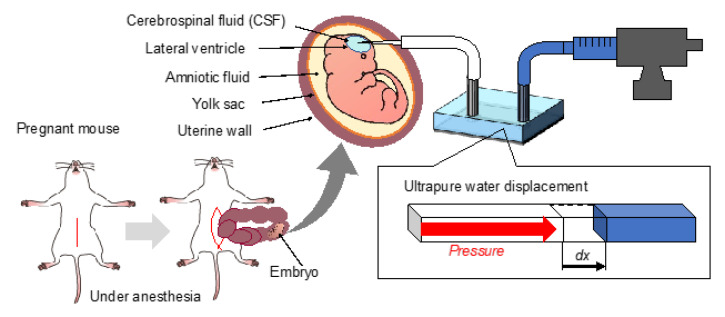
Preparation method used for measurements in mouse embryos. Under anesthesia, the abdomen of the pregnant mouse was opened to expose the uterus. The intraventricular pressure of the lateral ventricles of the mouse embryo was measured through the uterine wall. The environment surrounding the mouse embryo: The brain ventricles of the mouse embryo are filled with CSF, and the mouse embryo is situated in the amniotic fluid, surrounded by the yolk sac and the uterine wall.

The intraventricular pressure of mouse embryos during development was measured using the fabricated device. Figure 4 depicts the method of preparation of the mouse embryos for measurement. Pregnant mice were deeply anesthetized with a mixed anesthetic comprising midazolam and butorphanol. The mixture contained 4 mg/kg midazolam (Sandoz Inc., Basel, Switzerland), 0.75 mg/kg medetomidine (Nippon Zenyaku Kogyo Co., Ltd., Tokyo, Japan), and 5 mg/kg butorphanol tartrate (Meiji Seika Pharma Co., Ltd., Tokyo, Japan) to allow live-state measurements. The abdomen of each anesthetized pregnant mouse was opened to expose the uterus. Mouse embryos were enclosed within the uterine wall and amniotic fluid, with embryonic ventricles filled with CSF. Intraventricular pressure was measured by directly inserting a capillary from outside the uterine wall into the lateral ventricles. The pressure was calculated based on the displacement of water during the measurement.

### 2.6. Summary of Statistical Analysis

The normality of the data was assessed using the Shapiro–Wilk test, and the homogeneity of variance was evaluated using the F-test, both with a significance level of 5%. As normality and homogeneity of variance were confirmed, a two-sample t-test assuming equal variances was performed to compare the means between the two groups.

## 3. Results and Discussion

### 3.1. Fabricated Microdifferential Pressure Measurement Device

**Figure 5:**
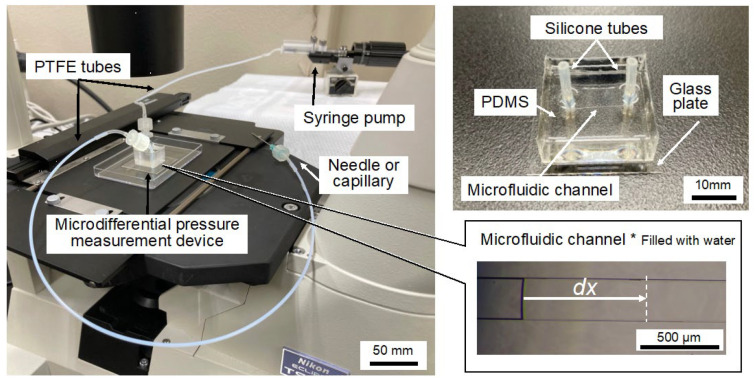
Fabricated microdifferential pressure measurement device. A syringe pump and either a needle or a capillary are connected through tubes attached to both ends of the microdifferential pressure measurement device. The displacement of the water within the microchannel is observed under an inverted microscope or a stereomicroscope.

The fabricated microdifferential pressure measurement device is shown in Figure 5. The microdifferential pressure measurement device is connected to a PTFE tube, which is further linked to a microsyringe pump and either a needle or a capillary at its tip. The device consists of a PDMS structure containing a microchannel positioned on a glass substrate. Silicone tubes are vertically connected to both ends of the microchannel. The microchannel is filled with ultrapure water. By observing the microchannel using a stereomicroscope or an inverted microscope, the displacement *dx* of the liquid surface caused by pressure can be confirmed.

### 3.2. Calibration Results for the Microdifferential Pressure Measurement Device

#### 3.2.1. Evaluation of the Glass Capillary Force

**Figure 6:**
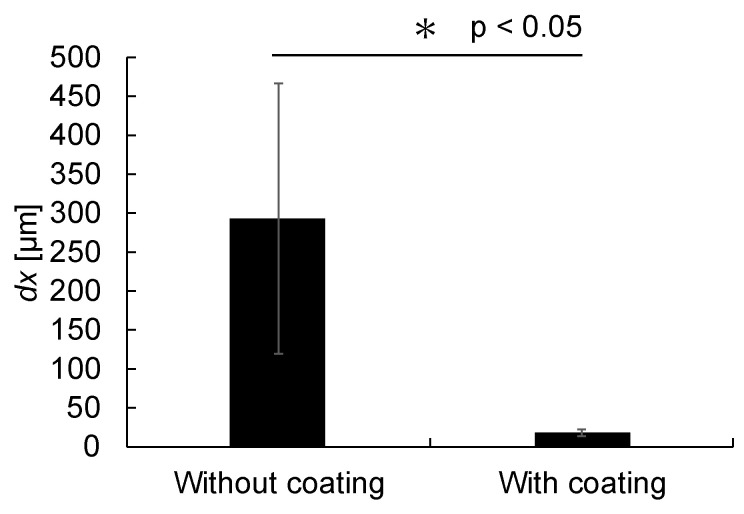
Displacement of ultrapure water liquid surface in the microchannel with and without a water-repellent coating. The displacements of ultrapure water in the microchannel for the glass capillary without and with a water-repellent coating were 293 ± 174 µm (*n* = 6) and 19 ± 5 µm (*n* = 6), respectively.

An immersion experiment was conducted using capillaries with and without water-repellent treatment to clarify the effect of capillary action when using a capillary. In the experiment, the tip of a glass capillary with an inner diameter of 100 µm was immersed in ultrapure water. The results of the displacement of ultrapure water in the microchannel when the capillary tip was immersed in the liquid are shown in Figure 6. The experiment lasted 300 s. The displacement of ultrapure water in the microchannel was 293 ± 174 µm (*n* = 6) for the capillary without water-repellent treatment and 19 ± 5 µm (*n* = 6) for the capillary with water-repellent treatment. These results showed that the capillary with water-repellent treatment had significantly less influence from capillary action (*p* = 0.0032, *t*-test) and exhibited a smaller standard deviation. Since pressure is calculated from displacement, the standard deviation of displacement directly affects the pressure. Therefore, we used the water-repellent-treated capillary in subsequent experiments to minimize this influence.

#### 3.2.2. Pressure Application Experiment Using a Capillary

**Figure 7:**
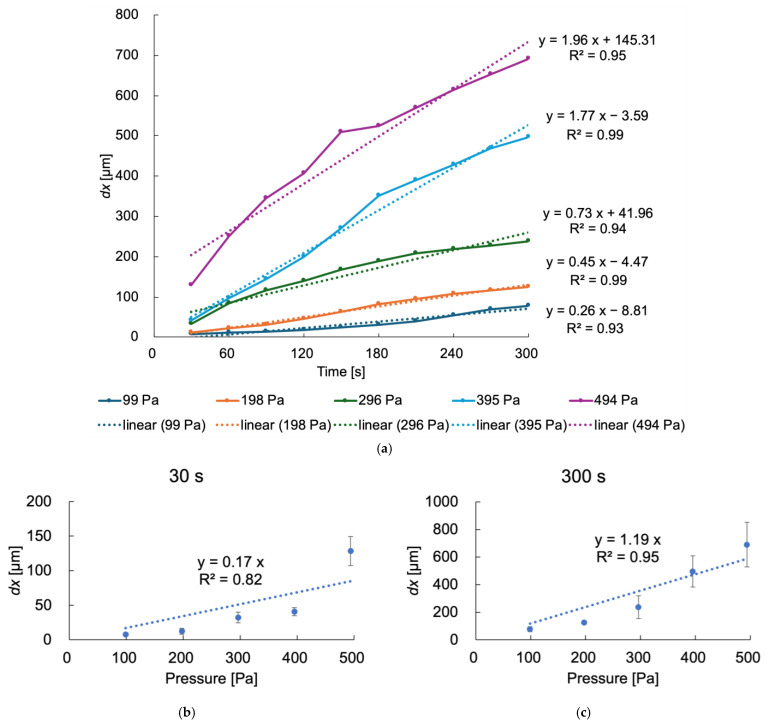
(**a**) Relationships between time and ultrapure water displacement in the device at different pressures; pressure was applied by changing the position of the capillary tip within FBS. (**b**) Relationships between pressure and ultrapure water displacement at 30 s. (**c**) Relationships between pressure and ultrapure water displacement at 300 s.

To calibrate the microdifferential pressure measurement device, considering the influence of the capillaries, pressure was applied to the device by immersing the tip of the capillary in FBS from 0 mm to 50 mm. Based on the results from Figure 6, the water-repellent-treated capillary, which had a smaller standard deviation, was used. The results of the pressure application experiment are shown in Figure 7. The displacement *dx* was measured at 30 s intervals from 0 to 300 s. As the pressure increased, the displacement *dx* also increased, and it continued to grow over time. When the pressure applied to the device increased, the displacement *dx* of ultrapure water in the microchannel increased. The displacement of ultrapure water increased as time passed, with a correlation coefficient of more than 0.93, indicating a linear trend. The relationship between the displacement of the liquid surface in the microchannel and the pressure at each time point was obtained using a linear regression of pressure and displacement. As a representative example, the relationships between pressure and displacement at 30 s and 300 s are shown in Figure 7b and Figure 7c, respectively. Three measurements (*n* = 3) were conducted at each pressure. Regression equations at each time point were used to derive the constant c_1_ and the coefficient of determination R^2^, summarized in Table 1. As time progressed, c_1_ increased, and R^2^ reached a maximum of 0.95. The maximum standard error was 93 Pa. It was found that the longer the measurement time, the higher the R^2^ value.

#### 3.2.3. Calculation Results for the Glass Capillary Force

The relationship between the liquid surface displacement *dx*, pressure *P*, and constant c was determined through the calibration described earlier. As explained, pressure was applied by immersing the tip of a water-repellent-treated capillary in FBS and measuring the displacement *dx* over time at intervals of 30 s. Linear regression was used to analyze the relationship between pressure and displacement at each time point, including the 300 s mark. At 300 s, the slope of the linear regression equation provided the constant c_1_, which was calculated to be 1.19, as shown in Figure 7c and Table 1. This value corresponds to the relationship between the displacement *dx* [µm] of ultrapure water and the pressure *P* [Pa], as expressed by Equation (3). The coefficient of determination was 0.95.

**Figure 8:**
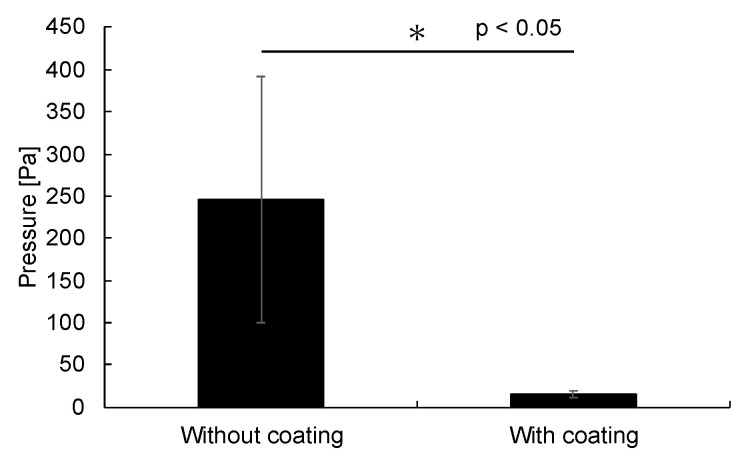
Calculation results for the glass capillary force. The pressures for the capillaries without and with a water-repellent coating were 246 ± 146 Pa (*n* = 6) and 16 ± 4 Pa (*n* = 6).

(3) dx=1.19P,

The results of the pressure calculation from this equation are shown in Figure 8. The pressures for the capillary without and with water-repellent treatment were 246 ± 146 Pa (*n* = 6) and 16 ± 4 Pa (*n* = 6), respectively. In this experiment, the tip of the capillary (1 to 3 mm) was immersed in ultrapure water; if capillary action was not present, the pressure should have been in the range of 10–30 Pa. Therefore, based on the experimental results, it is believed that a capillary force of approximately 100–200 Pa was acting in the capillary without water-repellent treatment. In the capillary with water-repellent treatment, the pressure was approximately 10 Pa and fell within the 10–30 Pa range, indicating that capillary action was suppressed (*p* = 0.0032, *t*-test). Additionally, the standard deviation was also smaller than that of the capillary without water-repellent treatment. From these results, it was determined that even if the test solution was ultrapure water instead of FBS, there would be no impact on the pressure, as the measured pressure remained the same as the applied pressure.

### 3.3. Lateral Ventricular Pressure Measurements in Mouse Embryos

**Figure 9:**
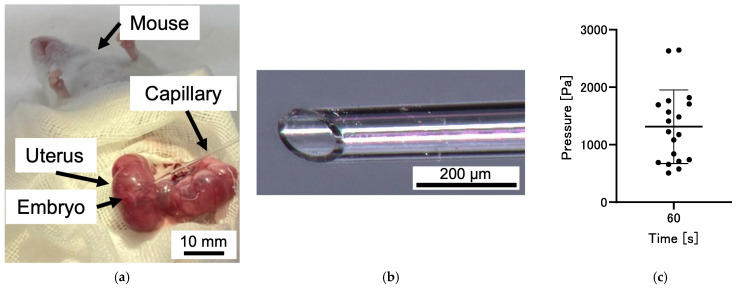
(**a**) The tip of the glass capillary was polished to a 45-degree angle with an inner diameter of 100 µm. (**b**) Overview of intraventricular pressure measured in mouse embryos. (**c**) Ventricular pressure in E12.5 mouse embryos: 1313 ± 640 Pa (mean ± standard deviation, *n* = 19).

Experiments were conducted to demonstrate the capability of the device to measure intraventricular pressure in mouse embryos during their gestational period. Mouse embryos at developmental stage E12.5, a stage where capillary insertion could be visually confirmed, were selected for the study. Figure 9a shows the process of intraventricular pressure measurement in the mouse embryos. Pregnant mice were anesthetized, and the uterus was exposed. The capillary was inserted into the lateral ventricles of the mouse embryos in utero. A water-repellent-treated glass capillary was used for the measurements. The tip of the capillary was polished to a 45-degree angle to ensure smooth insertion into the tissue. The appearance of the capillary tip is shown in Figure 9b. Once inserted, the intraventricular pressure displaced ultrapure water in the microchannel toward the injection port. The displacement of water in the microchannel is illustrated in Supplementary Materials Video S1. As it was difficult to insert the capillary into the embryo’s brain ventricle through the uterine wall using a manipulator, the insertion was performed manually. To minimize the influence of uterine contractions, the measurement time was shortened. However, with a measurement time of 30 s, the coefficient of determination remained at 0.82, as shown in Table 1, indicating insufficient linearity in the calibration curve. Therefore, the measurement time was adjusted to 60 s to ensure linearity. In total, 19 mouse embryos were measured. The displacement of ultrapure water reached values between 183 and 952 µm at the 60 s mark. The pressure was calculated using Equation (4), derived from the capillary with water-repellent treatment at the 60 s point. From Figure 7 and Table 1, the constant c_1_ was determined to be 0.36, with a coefficient of determination of 0.87. The maximum error at the 60 s measurement point was 23 Pa.

(4) dx=0.36 P,

Figure 9c shows that the pressure ranged from 508 to 2645 Pa, with an average of 1313 Pa and a standard deviation of 640 Pa (*n* = 19). Previous reports indicated that the pressure exerted on the head of a mouse embryo without the uterine wall at E13 was in the range of 77–93 Pa [33], and the intracranial pressure in newborn mice was 177 ± 116 Pa (mean ± standard deviation) after 3 days and 256 ± 104 Pa after 10 days [34]. It has been reported that the intrauterine pressure in mice is approximately 1053 Pa (7.9 mmHg) at E12.5 [35]. Moreover, a recently reported preprint estimated the pressure to be ~1400 Pa [36]. The measured results from this experiment were close to these values, indicating that the obtained values are reasonable.

The considerably large standard deviations observed in both the measured and literature values can be attributed to individual differences in animal embryos. However, as the measurement target is ultrasmall and minute in volume, small external environmental noise may also have a significant impact. This could represent the current technological limit. Existing sensors for pressure measurement have several limitations when applied to microscale environments. Many conventional sensors have been designed for underwater measurements and are dependent on electrical properties, making them susceptible to external factors such as media ion composition, electrical resistance, and ionic diffusion. In contrast, the proposed device overcomes these limitations by operating effectively in the air and eliminating reliance on electrical measurements. The proposed device achieved the micropressure measurement in microscale spaces embedded in multilayered tissues by exploiting the displacement of ultrapure water in the microchannel for minimizing the effect of external factors.

This performance is comparable to previously reported methods, such as a microscale pressure measurement method based on the curvature deformation of immiscible fluid/fluid interfaces [29], piezoresistive sensors [34], and servo-null sensors [25], which demonstrate a resolution of less than 25 Pa. The proposed device achieved a maximum standard error of 23 Pa at the 60 s mark, indicating equivalent performance. However, with prolonged use, the maximum standard error increased to 93 Pa, highlighting a key limitation that will need to be addressed in future iterations.

Microenvironmental pressure measurements face several limitations that need to be addressed to improve the accuracy and reliability of the device: 1. Validation challenges in microenvironments: Direct validation using microenvironments with known pressures remains technically challenging. While the current device accounts for factors such as air compression, silicone tube deformation, and frictional forces during calibration, creating a stable reference environment with precisely known pressures is difficult. Future work should focus on developing calibration systems that enable more precise pressure control in microspace. 2. Influence of external noise: Measurements in microenvironments are highly susceptible to external environmental noise, including small vibrations, temperature fluctuations, and humidity changes. Such noise can significantly affect the accuracy of the results. Developing systems that minimize these external influences is crucial for improving measurement reliability. 3. System complexity and material limitations: The elastic deformation of components, such as the microchannel and connecting tubes, introduces variability. Although these factors are accounted for in calibration, eliminating their effects remains challenging. Furthermore, the materials used, such as PDMS and silicone, may experience degradation or instability during prolonged use, potentially affecting measurement accuracy. 4. Manual capillary insertion: Currently, the capillary insertion into the lateral ventricles of mouse embryos is performed manually, which introduces variability and limits reproducibility. Automating this process using robotic or manipulator systems could improve consistency and reduce operator-dependent errors. However, inserting a capillary into the lateral ventricles of an unfixed embryo within amniotic fluid poses significant challenges.

These limitations highlight areas where further development is necessary to enhance the reliability and precision of the device. Addressing these challenges will not only improve current measurement techniques but also pave the way for broader applications in developmental biology and medical diagnostics.

### 3.4. Discussion of Measurement Results

In this device, the length of the PTFE tubes was set to 400 mm. Using excessive amounts of compressible air at the distal end may not be suitable for measuring pressure at that point. Therefore, we theoretically calculated the amount of air compression in a 400 mm PTFE tube using the equation for compressibility:

(5) $$\beta =-\frac{1}{V}\frac{dV}{dP},$$

where *β* is the compressibility of the fluid, *V* is the volume of the fluid, *dV* is the change in volume of the fluid, and *dP* is the change in pressure.

As the cross-sectional area of the flow channel is constant, the change in volume *dV* in Equation (5) can be rewritten using the cross-sectional area of the flow channel *A* and the displacement of the fluid *dx* (see Equation (6)):

(6) $$\beta =-\frac{1}{V}\frac{A\cdot dx}{dP},$$

where *A* is the cross-sectional area of the flow channel, and *dx* is the displacement of the fluid. By rearranging Equation (6) in terms of pressure *dP*, we can obtain Equation (7).

(7) $$dP=-\frac{A\cdot dx}{\beta \cdot V},$$

Using a PTFE tube with a length of 400 mm and an inner diameter of 0.5 mm and assuming that a pressure of 100 Pa is applied, substituting *dP* = 100 Pa, *V* = 78.5 mm^3^, *A* = 0.20 mm^2^, and the compressibility of air *β* = 9.87 × 10^−6^ Pa^−1^ (at 20 °C, 1 atm) into Equation (7), the compression length of air inside the PTFE tube per 100 Pa was calculated to be 387 µm. Furthermore, when considering the displacement within the microchannel, based on a cross-sectional area of 0.025 mm^2^, the displacement was estimated to be 3.10 mm. From Figure 7, displacement is observed even at the 30 s mark at a pressure of 100 Pa, indicating that the air compression within the 400 mm PTFE tube is within acceptable limits. Adjusting the PTFE tube length provides a means to influence the measurement time and pressure-sensing range of the device. Shortening the PTFE tube reduces the air volume inside, decreasing its compressibility. This results in faster response times, quicker stabilization, and a reduction in overall measurement time. Additionally, the reduced air volume enhances the displacement of the liquid surface under pressure, thereby improving sensitivity. However, the finite length of the microchannel imposes a limit on the measurement range. Conversely, increasing the length of the PTFE tube increases the air volume and its compressibility. This leads to longer stabilization times, as the system requires more time to achieve uniform compression. While the displacement of the liquid surface decreases under these conditions, the pressure range of the device expands accordingly. Thus, by appropriately adjusting the PTFE tube length, the measurement time and pressure-sensing range of the device can be modified. This enables the optimization of the device’s performance to suit the specific requirements of the sample and experimental conditions.

In this device, 1 mL of ultrapure water was used. The compression volume was theoretically calculated to determine the extent to which the water is compressed by pressure. Assuming a pressure of 1000 Pa is applied, with *dP* = 1000 Pa, *V* = 1 mL, microchannel cross-sectional area *A* = 0.025 mm^2^, and water compressibility *β* = 4.5 × 10^−10^ Pa^−1^ (at 20 °C, 1 atm), substituting these values into Equation (7) calculated the compression length of water at 1000 Pa to be 18 μm.

The device uses PDMS to construct the microchannel and a silicone tube to connect the microchannel with the PTFE tube. The microchannel has a width of 250 µm and a height of 100 µm, with a PDMS thickness of over 10 mm, so deformation of the PDMS was considered negligible. Therefore, the deformation of the silicone tube was evaluated here. The circumferential stress *σ_θ_* is given by Equation (8):

(8) $$\sigma_{\theta }=\frac{pr}{t},$$

*p* represents the internal pressure, *r* is the radial coordinate, and *t* is the wall thickness.

According to Hooke’s law,

(9) $$\varepsilon =\frac{\sigma_{\theta }}{E},$$

*ε* represents the strain, and *E* is the Young’s modulus.

Substituting Equation (8) into Equation (9), the following equation was obtained:

(10) $$\varepsilon =\frac{pr}{Et},$$

Here, Young’s modulus of the silicone tube was set to 2 MPa [37], based on the Shore A hardness of 56 for the silicone tube (9-869-02, AS ONE Corp.). The tube has an outer diameter of 2 mm and an inner diameter of 1 mm, resulting in a wall thickness (*t*) of 0.5 mm and a radius (*r*) of 0.5 mm. When a pressure of 1000 Pa is applied, the strain (*ε*), calculated from Equation (10), is 5 × 10^−4^ or 0.05%. Although each silicone tube has a length of 15 mm, half of it is embedded in the PDMS, making the effective length 7.5 mm. Converting the deformation of the silicone tube into volume and subsequently into displacement within the microchannel, the resulting displacement is 353 µm.

This analysis indicates that the displacement of water within the microchannel is influenced more significantly by the elastic deformation of the silicone tube than by the compression of the water itself. Additionally, when a pressure of 1000 Pa is applied to the device, the actual pressure exerted on the water-side silicone tube is likely less than 1000 Pa. This reduction is due to pressure absorption caused by air compression within the device, the silicone tube’s deformation on the air side, and frictional forces acting on the water. The displacement of water within the microchannel depends primarily on the elastic deformation of the microchannel and connecting tube. Therefore, material selection plays a critical role in adjusting the pressure range and sensitivity of the device. For elastic materials such as PDMS or silicone, choosing materials with a lower Young’s modulus allows deformation under minimal pressure, improving sensitivity. However, excessive deformation may make high-pressure measurements challenging. In contrast, rigid materials like glass or hard plastics can suppress deformation, enhancing accuracy in high-pressure ranges, but at the cost of reduced sensitivity in lower-pressure ranges. If deformation occurs in the microchannel, it may impact the measurement of liquid surface displacement. To address this, it is preferable to select materials for the microchannel that are resistant to deformation. Alternatively, as implemented in this device, increasing the wall thickness of the microchannel can effectively suppress deformation. This approach ensures more accurate measurements and enhances the overall reliability of the device. Ultimately, the careful consideration of material properties and structural design is essential for achieving a balance between sensitivity, measurement range, and reliability, enabling the device to perform optimally for a wide range of applications.

## 4. Conclusions

In this study, we introduced a microdifferential pressure measurement device capable of measuring pressure at the cellular scale while remaining unaffected by factors such as media ion composition, environmental electrical resistance, and ionic diffusion characteristics. This method operates in the air without the need for electrical measurements, relying solely on the displacement of ultrapure water within the microchannel. Moreover, the fabrication of the device is cost-effective and straightforward. The performance of the developed device was assessed by measuring the intraventricular pressure in mouse embryos during development. The measured intraventricular pressure was 1313 ± 640 Pa (mean ± standard deviation), a result in line with findings from previous studies. Thus, the device successfully demonstrated the measurement of pressure within microscale spaces embedded in multilayered tissues that have conditions similar to those of the real biological environment. By eliminating the need for water immersion and functioning effectively in air, this device addresses the major limitations of conventional methods and expands the scope of pressure measurement technologies.

The results highlight the versatility of the device across various types of animal embryos and its potential for a broad range of applications, including the measurement of minute pressures within living organisms or in other fields requiring precise and localized pressure measurements. Moreover, the device can be useful for a broad range of research and clinical settings in biomechanics, developmental biology, and potential pathophysiological investigations where microenvironmental pressures are relevant. The adaptability, simplicity, and efficiency of the device can advance the understanding and manipulation of mechanical forces at the cellular and tissue levels.

This study demonstrated the feasibility and versatility of the microdifferential pressure measurement device, but several challenges remain to enhance its applicability and performance. Key issues include calibration in controlled microenvironments, automation of capillary insertion, expansion to broader biological applications, and adaptation for dynamic pressure measurements. Addressing these challenges will improve the device’s accuracy and reliability, paving the way for applications in developmental biology, medical diagnostics, and other fields. The findings of this study provide a foundation for further improvements and broader applications in the future.

## Figures and Tables

**Table 1:** Relationship between constant *c*_1_, the coefficient of determination R^2^, and the maximum standard error S.E. _max_ at each time point.

Time [s]	*c*_1_	R^2^	S.E. _max_
30	0.17	0.82	12
60	0.36	0.87	23
90	0.50	0.87	44
120	0.62	0.90	55
150	0.79	0.91	84
180	0.88	0.94	64
210	0.97	0.95	70
240	1.05	0.95	81
270	1.13	0.95	89
300	1.19	0.95	93

## Data Availability

Data from the experiments can be available upon request.

## Supplementary Materials

The following supporting information can be downloaded at: https://www.mdpi.com/article/10.3390/bioengineering12010003/s1. Video S1: The displacement of water within the microchannel.

## References

[1] Gudipaty S.A., Lindblom J., Loftus P.D., Redd M.J., Edes K., Davey C.F., Krishnegowda V., Rosenblatt J. (2017). Mechanical stretch triggers rapid epithelial cell division through Piezo1. Nature.

[2] Kureel S.K., Sinha S., Purkayastha P., Barretto S., Majumder A. (2022). Substrate Stiffness Controls the Cell Cycle of Human Mesenchymal Stem Cells Via Cellular Traction. JOM.

[3] Uroz M., Wistorf S., Serra-Picamal X., Conte V., Sales-Pardo M., Roca-Cusachs P., Guimera R., Trepat X. (2018). Regulation of cell cycle progression by cell-cell and cell-matrix forces. Nat. Cell Biol..

[4] Miroshnikova Y.A., Le H.Q., Schneider D., Thalheim T., Rubsam M., Bremicker N., Polleux J., Kamprad N., Tarantola M., Wang I. (2018). Adhesion forces and cortical tension couple cell proliferation and differentiation to drive epidermal stratification. Nat. Cell Biol..

[5] Reilly G.C., Engler A.J. (2010). Intrinsic extracellular matrix properties regulate stem cell differentiation. J. Biomech..

[6] Heisenberg C.P., Bellaiche Y. (2013). Forces in tissue morphogenesis and patterning. Cell.

[7] Krieg M., Arboleda-Estudillo Y., Puech P.H., Kafer J., Graner F., Muller D.J., Heisenberg C.P. (2008). Tensile forces govern germ-layer organization in zebrafish. Nat. Cell Biol..

[8] Petridou N.I., Grigolon S., Salbreux G., Hannezo E., Heisenberg C.P. (2019). Fluidization-mediated tissue spreading by mitotic cell rounding and non-canonical Wnt signalling. Nat. Cell Biol..

[9] Brassard J.A., Lutolf M.P. (2019). Engineering Stem Cell Self-organization to Build Better Organoids. Cell Stem Cell.

[10] Drakhlis L., Biswanath S., Farr C.M., Lupanow V., Teske J., Ritzenhoff K., Franke A., Manstein F., Bolesani E., Kempf H. (2021). Human heart-forming organoids recapitulate early heart and foregut development. Nat. Biotechnol..

[11] Campas O., Mammoto T., Hasso S., Sperling R.A., O’Connell D., Bischof A.G., Maas R., Weitz D.A., Mahadevan L., Ingber D.E. (2014). Quantifying cell-generated mechanical forces within living embryonic tissues. Nat. Methods.

[12] Mohagheghian E., Luo J., Chen J., Chaudhary G., Chen J., Sun J., Ewoldt R.H., Wang N. (2018). Quantifying compressive forces between living cell layers and within tissues using elastic round microgels. Nat. Commun..

[13] Etournay R., Popovic M., Merkel M., Nandi A., Blasse C., Aigouy B., Brandl H., Myers G., Salbreux G., Julicher F. (2015). Interplay of cell dynamics and epithelial tension during morphogenesis of the Drosophila pupal wing. Elife.

[14] Fernandez-Gonzalez R., Zallen J.A. (2013). Wounded cells drive rapid epidermal repair in the early Drosophila embryo. Mol. Biol. Cell.

[15] Liang X., Michael M., Gomez G.A. (2016). Measurement of Mechanical Tension at Cell-cell Junctions Using Two-photon Laser Ablation. Bio-Protoc..

[16] du Roure O., Saez A., Buguin A., Austin R.H., Chavrier P., Silberzan P., Ladoux B. (2005). Force mapping in epithelial cell migration. Proc. Natl. Acad. Sci. USA.

[17] Liu Z., Tan J.L., Cohen D.M., Yang M.T., Sniadecki N.J., Ruiz S.A., Nelson C.M., Chen C.S. (2010). Mechanical tugging force regulates the size of cell-cell junctions. Proc. Natl. Acad. Sci. USA.

[18] Lekka M., Gnanachandran K., Kubiak A., Zielinski T., Zemla J. (2021). Traction force microscopy—Measuring the forces exerted by cells. Micron.

[19] Brugues J., Maugis B., Casademunt J., Nassoy P., Amblard F., Sens P. (2010). Dynamical organization of the cytoskeletal cortex probed by micropipette aspiration. Proc. Natl. Acad. Sci. USA.

[20] Evans E., Yeung A. (1989). Apparent viscosity and cortical tension of blood granulocytes determined by micropipet aspiration. Biophys. J..

[21] Yeung A., Evans E. (1989). Cortical shell-liquid core model for passive flow of liquid-like spherical cells into micropipets. Biophys. J..

[22] Beauzamy L., Derr J., Boudaoud A. (2015). Quantifying hydrostatic pressure in plant cells by using indentation with an atomic force microscope. Biophys. J..

[23] Li M., Zhang C., Wang L., Liu L., Xi N., Wang Y., Dong Z. (2013). Investigating the morphology and mechanical properties of blastomeres with atomic force microscopy. Surf. Interface Anal..

[24] Desmond M.E., Levitan M.L., Haas A.R. (2005). Internal luminal pressure during early chick embryonic brain growth: Descriptive and empirical observations. Anat. Rec. Part A Discov. Mol. Cell. Evol. Biol..

[25] Fein H. (1972). Microdimensional pressure measurements in electrolytes. J. Appl. Physiol..

[26] Fox J.R., Wiederhielm C.A. (1973). Characteristics of the servo-controlled micropipet pressure system. Microvasc. Res..

[27] Petrie R.J., Koo H. (2014). Direct measurement of intracellular pressure. Curr. Protoc. Cell Biol..

[28] Petrie R.J., Koo H., Yamada K.M. (2014). Generation of compartmentalized pressure by a nuclear piston governs cell motility in a 3D matrix. Science.

[29] Yang J., Duan X., Fraser A.K., Choudhury M.I., Ewald A.J., Li R., Sun S.X. (2019). Microscale pressure measurements based on an immiscible fluid/fluid interface. Sci. Rep..

[30] Eddington D.T., Puccinelli J.P., Beebe D.J. (2006). Thermal aging and reduced hydrophobic recovery of polydimethylsiloxane. Sens. Actuators B Chem..

[31] Shinohara H., Mizuno J., Shoji S. (2007). Low-temperature direct bonding of poly(methyl methacrylate) for polymer microchips. IEEJ Trans. Electr. Electron. Eng..

[32] Wonerow T., Uhler M., Nuppnau J., Kretzer J.P., Mantwill F. (2021). Rheologic Behavior of Bovine Calf Serum. Materials.

[33] Tsujikawa K., Saito K., Nagasaka A., Miyata T. (2022). Developmentally interdependent stretcher-compressor relationship between the embryonic brain and the surrounding scalp in the preosteogenic head. Dev. Dyn..

[34] Moazen M., Alazmani A., Rafferty K., Liu Z.J., Gustafson J., Cunningham M.L., Fagan M.J., Herring S.W. (2016). Intracranial pressure changes during mouse development. J. Biomech..

[35] McCafferty R.E., Wood M.L., Knisely W.H. (1964). Uterine Contractions and Intra-Amniotic Pressures in Gravid Mice. Am. J. Obstet. Gynecol..

[36] Tsujikawa K., Muramatsu R., Miyata T. (2024). CSF pressure in fetal mice in utero: External factors pressurize the intraventricular space. BioRxiv.

[37] Boyce M.C., Joyce K., Qi H.J. (2003). Durometer Hardness and the Stress-Strain Behavior of Elastomeric Materials. Rubber Chem. Technol..

